# Glucose Metabolism and Sex Hormones in Male Patients with Medication-naïve First-episode Schizophrenia: A Large-scale Cross-sectional Study

**DOI:** 10.2174/1570159X22666240212141602

**Published:** 2024-02-16

**Authors:** Meihong Xiu, Meng Hao, Cai Liu, Maodi Sun, Xiaoe Lang

**Affiliations:** 1 Peking University HuiLongGuan Clinical Medical School, Beijing Huilongguan Hospital, Beijing, China;; 2 Department of Psychiatry, First Hospital of Shanxi Medical University, Taiyuan, China;; 3 North University of China, Taiyuan, China

**Keywords:** Schizophrenia, first-episode, glucose metabolism, sex hormones, fasting insulin, male patients

## Abstract

**Background:**

Schizophrenia (SCZ) usually begins in early adult life. The underlying molecular mechanisms of SCZ remain unclear. There is evidence for the involvement of abnormalities in metabolic and endocrine systems in SCZ, even in drug-naïve first-episode schizophrenia patients (DNFES). However, the association between impaired regulation of glucose metabolism and sex hormones was not studied in SCZ. This study aimed to evaluate the interrelationship between sex hormones and high fasting glucose levels in male DNFES patients.

**Methods:**

A total of 99 patients with SCZ were recruited, and fasting glucose, fasting insulin, the insulin resistance index (HOMA-IR), and sex hormones were measured.

**Results:**

We found that some male patients with SCZ had abnormal levels in glucose metabolism parameters and gonadal hormones that were not within the normal range. Linear regression analysis adjusted for age, waist circumference, and body mass index showed that testosterone levels were negatively associated with fasting insulin in male patients (β = -0.21, t = -2.2, *p* = 0.03).

**Conclusion:**

Our findings confirm the abnormalities in glucose metabolism parameters and gonadal hormones at the onset of the illness in male DNFES patients with SCZ. In addition, there was an interaction effect between abnormal glucose metabolism and sex hormones in male patients.

## 1 INTRODUCTION

Schizophrenia (SCZ) is a major public health problem affecting 1% of the general population [1]. The exact pathophysiological mechanisms of SCZ remain unclear. Recent literature based on pharmacological studies and genetic research suggests that abnormalities in the neuroendocrine and cardiometabolic systems may interact with early neurodevelopmental defects associated with predisposing genetic aberrations in relation to the molecular etiology of SCZ [2, 3].

Many previous observational studies have reported that type 2 diabetes and other indices of metabolic dysregulation are common in patients with SCZ, resulting in a 15-20-year reduction in life expectancy due to the early onset of cardiometabolic disease and cardiovascular disease [4-9]. The prevalence of type 2 diabetes in the population with SCZ is 3-9 times higher than in the general population [10].

Accumulating data suggest that some components of metabolic syndrome are already present in the early psychosis of SCZ before antipsychotic treatment [11-13]. Indeed, evidence from antipsychotic-naïve patients with SCZ found the presence of a pre-diabetic state or impaired glucose metabolism and diabetes at the onset of SCZ [14, 15]. The higher prevalence of metabolic dysfunction in SCZ may be due to low socioeconomic status, disordered eating and lifestyle, and poor diet, as well as the presence of shared susceptibility genes between SCZ and type 2 diabetes [16, 17]. Insulin signaling has been shown to be involved in the mechanism of the link between SCZ and cardiovascular risk factors [18].

Notably, glucose metabolism and insulin signaling may be regulated by gonadal hormones [19, 20], with age-dependent effects [21-23]. Indeed, studies have found that plasma testosterone (TESTO) is directly associated with insulin sensitivity and that low TESTO levels are associated with an increased risk of type 2 diabetes [24]. In male rats, castration-induced TESTO deficiency significantly reduced insulin sensitivity and led to increased fasting glucose levels [25]. On the other hand, in post-menopausal women and ovariectomized animal models, loss of estrogen has been associated with central obesity, increased insulin resistance, and the development of complications such as type 2 diabetes [26]. The available data support that abnormal levels of gonadal hormones may increase the risk of SCZ development and worsen symptom severity [27, 28].

As abnormal glucose metabolism and endocrine dysfunction are involved in the pathogenesis of SCZ, there is a need to investigate the interrelationship between these abnormalities early in the onset stage of the disease after controlling some confounding factors, such as age, chronicity, antipsychotic medication, *etc*. On this basis, patients at onset are better suited to test the hypothesis of an intrinsic relationship between endocrine metabolic dysfunction and SCZ compared to chronic patients. Therefore, the purpose of this study was to extend the previous findings on abnormal glucose metabolism and gonadal hormones in patients with SCZ. We hypothesized that there is greater impairment of these markers in SCZ and that there is an interaction between glucose metabolism and sex hormones in SCZ. Therefore, this study would answer the following questions: 1) to conduct an evaluation of glucose metabolism and gonadal hormones in drug-naïve first-episode schizophrenia (DNFES) patients; and 2) to understand the interrelationship between glucose metabolism and sex hormones in male patients, adjusting for age, waist circumference and BMI.

## 2 METHODS

### 2.1 Samples

The study protocol was approved by the Ethics Committee of First Hospital of Shanxi Medical University (NO. 2018-K003), and written informed consent was obtained from the patients. We undertook this study between June 1, 2018, and May 31, 2021.

Ninety-nine patients were recruited and met the Diagnostic and Statistical Manual for Mental Disorders (DSM), Fourth Edition (DSM-IV), which was confirmed by two psychiatrists using the Structured Clinical Interview for DSM-IV-TR Axis I Disorders-Patient Edition (SCID-I/P). The SCID-I/P is a semi-structured interview guide for making diagnoses according to the diagnostic criteria published in the American Psychiatric Association’s DSM, which has been shown to be reliable and valid in China [29]. They were all drug-naïve first-episode patients with a duration of illness < 5 years based on a previous study [30]. The inclusion criteria included: 1) first episode of psychosis and medication-naïve status; 2) 18-45 years of age; 3) duration of illness not more than five years; and (4) male. Patients also need to meet the following exclusion criteria: (1) autism spectrum disorders; (2) mental retardation; (3) abuse or substance dependence; (4) diagnosable organic brain disease or major medical somatic comorbidities (*e.g*., cancer, diabetes, cerebrovascular disease, or hypertension); (5) any history of endocrine disorder; (6) signs of hyperandrogenism, such as acne and hirsutism; and (7) currently receiving hormone replacement therapy or hormonal contraceptives.

### 2.2 Measurements of Glucose Metabolism Parameters

Fasting venous blood samples (5 ml) were drawn from all patients into BD Vacutainer Rapid Serum Tubes between 7:30 AM and 8:30 AM and were centrifuged for 10 min at 4,000 g. Serum levels of glucose and gonadal hormone were quantified from fresh blood samples following centrifugation. The biochemical analysis was carried out in duplicate at the hospital laboratory by three technicians blind to the clinical status of the participants.

Serum glucose (FBG) levels were measured using an enzymatic method and by HITACHI 7600 automatic biochemistry analyzer (Hitachi, Japan). The normal range for an FBG level was 3.9-6.1 mmol/L in China. Serum insulin (FINS) levels and glycated hemoglobin (HbA1c) levels were measured using commercially available kits (Roche Diagnostics, GmbH, Mannheim, Germany). Insulin was analyzed on Cobas 6000 (Roche), and HbA1c was determined on the Roche Cobas 501. The normal ranges for the levels of insulin and HbA1c were from 2.6 to 24.9 μU/mL and from 4.8% to 5.9%, respectively. Insulin resistance index HOMA-IR was determined by using the homeostatic model of assessment (HOMA) as the product of fasting serum insulin levels and fasting serum glucose levels divided by 22.5.

### 2.3 Measurements of Gonadal Hormone Levels

Serum levels of sex hormone indicators, including estradiol (E2), follicular-stimulating hormone (FSH), progesterone (PROG), luteinizing hormone (LH) and TESTO, were measured using the chemiluminescence immunoassays by an automated Beckman UniCel DxI800A analyzer (Beckman Coulter, Brea, CA, USA). The normal ranges for FSH, PROG, LH, TESO and E2 were 1.27-19.26 mIU/ml, 0.45-6.55 nmol/L, 1.24-8.62 mIU/ml, 6.07-27.1 nmol/L and 14-55 pmol/L.

### 2.4 BMI and Waist Circumference Measurements

Weight and height were measured in a standardized fashion to calculate BMI (weight over squared height, Kg/m^2^). Height was measured to the nearest millimeter, with the subjects barefooted and standing upright. Weight was measured with an electronic scale, and patients were weighed in light indoor clothing.

Waist circumference was measured at the end of normal expiration at the umbilical waist (horizontal at the umbilicus).

### 2.5 Statistical Analysis

Statistical analysis was performed in SPSS, version 22.0. Statistical significance *p* was fixed to 0.05.

The categorical variable was reported as numbers with percentages, and the normally distributed numerical variable was reported as mean ± standard deviation. Nonnormally distributed numerical variable was reported with median and interquartile ranges (IQRs). Since most of the data were not normally distributed, non-parametric tests were generally used in this study.

Spearman-Rho correlation analysis was performed for the calculation of correlations between glucose metabolism parameters and gonadal hormones in male patients. Further linear regression analyses were used to confirm the association between these parameters after controlling for age, waist circumference and BMI. The Bonferroni corrections were conducted to adjust for multiple comparisons.

## 3 RESULTS

The demographic, glucose metabolism parameters, sex hormone levels and the normal reference ranges are shown in Table **1**. We identified the number of patients with abnormal glucose metabolism parameters as follows: n = 1 for HbA1c, n = 6 for FINS and n = 42 for HOMA-R. In addition, the number of patients who had abnormal sex hormone levels was n = 26 for E2 and n = 3 for LH.

Correlation analysis showed significant associations between age or onset age and FBG (r = 0.28, *p* = 0.005; r = 0.28, *p* = 0.006), between waist circumference and fasting FINS (r = 0.44, *p <* 0.001) and HOMA-IR (r = 0.45, *p <* 0.001), as well as between BMI and fasting FINS (r = 0.45, *p <* 0.001) and HOMA-IR (r = 0.44, *p <* 0.001) in male patients with SCZ.

Spearman correlation analysis showed that there were significant negative correlations of TESTO levels with FBG (r = -0.23, *p* = 0.025), FINS (r = -0.34, *p <* 0.001), and HOMA-IR (r = -0.35, *p <* 0.001) in male patients (Table **2**). In addition, PROG levels were negatively associated with FINS (r = -0.29, *p* = 0.004) and HOMA-IR (r = -0.28, *p* = 0.005). After Bonferroni corrections, the associations between FBG and HOMA-IR, as well as FINS, were statistically significant (all p_Bonferroni_ < 0.05). Moreover, regression analysis also supported the association after controlling for age, BMI, and waist circumference and found that TESTO levels were significantly associated with fasting FINS levels in male patients (β = -0.21, t = -2.2, *p* = 0.03) but not with PROG (β = -0.08, t = -0.84, *p* = 0.40) (Table **3**).

## 4 DISCUSSION

We found male DNFES patients with SCZ had abnormal levels of glucose metabolism parameters and sex hormones in the early stage of the disease. Additionally, in male patients, TESTO levels were negatively correlated with fasting insulin levels and HOMA-IR. To our present knowledge, it is the first study to explore the interrelationship between glucose metabolism and hormones in a large-scale sample of male DNFES patients in China.

Our findings of abnormal glucose metabolism parameters and sex hormone levels in male DNFES patients with SCZ were consistent with previous studies [31-33]. In particular, we found more patients with abnormal levels of fasting FINS and higher HOMA-IR. Insulin receptors have been reported to be widely distributed in the hippocampus, cerebral cortex, and hypothalamus [34]. Insulin resistance was found to be significantly associated with polygenic risk for SCZ, independent of age, ethnicity, lifestyle, and clinical factors [35]. When peripheral insulin levels are elevated, insulin levels in the cerebrospinal fluid are increased through the blood-brain barrier, which may lead to insulin resistance in the brain [36]. In addition, functional reductions in insulin signaling were also observed in patients with SCZ using the post-mortem brain [37, 38]. Recent data has also demonstrated impaired glia or neuronal insulin signal transduction in extracellular vesicles from the brain in SCZ [39, 40], as well as deleterious effects of impaired glucose/lactate supply on cerebral connectivity and myelinization [41-43].

Our study also demonstrates a disturbance of sex hormones, such as E2 and LH, in some male patients with SCZ. During adolescence, there are dramatic changes in the activation and amplification of the pulsed release of LH in boys and a rapid increase in circulating E2 [44]. The estrogen hypothesis postulates a protective role of E2 on the development and increased severity of SCZ [28, 45]. Estrogens have direct neuroprotective actions by impacting the levels of major neurotransmitter systems relevant to SCZ, such as dopamine signaling, glutamatergic, and serotonergic systems, as well as the density of receptors for these neurotransmitter systems [46-49]. Furthermore, hippocampal neurogenesis, such as the formation of dendritic spines and new neuronal connections, also depends on E2 levels in the hippocampus [50]. The density of dendritic spines in the hippocampus alters throughout the menstrual cycle, with the highest density occurring when E2 peaks [51]. Thus, when E2 levels are disturbed, it may lead to alterations in brain structure, including the number of neurons, neural plasticity and modifications in the bioenergetics of the brain. Recent studies have also reported that E2 deprivation induces mitochondrial dysfunctions and hippocampal synapse disruption in the brain, which may induce the decline of cognitions [52, 53] and further contribute to clinical symptoms in patients with SCZ.

This study also found that fasting insulin levels, insulin resistance index, and TESTO levels were negatively correlated in DNFES patients. The role of TESTO in the pathogenesis of SCZ is much less understood than the effects of estrogens. There was evidence that TESTO levels were lower in male patients with SCZ compared to sex- and age-matched controls [27, 54]. Under normal physiological conditions, TESTO plays a fundamental role in the maintenance of insulin sensitivity, insulin concentrations and islet β-cell function in men [24]. Studies in cultured adipocyte cells found that TESTO increased the phosphorylation of insulin receptor substrates-1 (Chen *et al.* 2006). Additionally, TESTO activates the cAMP-dependent protein kinase pathway by binding to the receptors in pancreatic islet β-cells and enhancing the regulation of the receptors by glucagon-like peptide-1 [55]. In preclinical studies, chronic high-calorie diet-induced obese male mice developed peripheral insulin resistance through increased adipose tissue accumulation and reduced blood TESTO levels [56-59]. Studies from men with hypogonadism and low TESTO levels have shown that insulin resistance develops over time, although patients do not show insulin resistance at the onset [60]. In particular, a retrospective study of 150 obese men by Souteiro *et al.* reported that 52% of patients were TESTO deficient and that their TESTO levels were negatively correlated with the insulin resistance index [61]. In the brain, studies show that TESTO plays a crucial role in brain development by influencing synaptic connectivity and neuronal differentiation [62, 63]. More importantly, TESTO also influences dopaminergic, glutamatergic and GABAergic neurotransmission systems, which contribute to the pathophysiology of SCZ [62]. All these studies support that abnormal TESTO signaling may serve as a risk factor for disturbed glucose metabolism and psychotic symptoms in male patients with SCZ.

Notably, although some patients with SCZ in our present study were abnormal in the glucose metabolism parameters and gonadal hormone levels, however, the average or median values of these biomarkers were within normal ranges. These findings that these biomarkers were not altered in patients are consistent with previous studies [31, 32], possibly because all patients were young patients in their first episode, with a median age of the patients 24 years.

This study had several limitations. First, the cross-section design limited the information available to infer the causal relationship between glucose metabolism parameters and hormones. Further longitudinal follow-up studies, including a large sample of DNFES patients are warranted to address this issue in the future by measuring hormone levels at appropriate times of the day and longitudinal evaluation in a substantial number of patients with SCZ. Second, we did not recruit a normal control group, and the comparisons with normal controls would give more information on our results. However, we could compare the levels of these biomarkers with the normal reference range in the general population. Third, clinical symptoms were not assessed using the Positive and Negative Syndrome Scales (PANSS), and we cannot analyze the associations between the severity of symptoms and insulin resistance. Additionally, the effects of diet and lifestyle on the relationship between glucose metabolism and sex hormones were not included in the statistical analyses. These data needed to be collected in a future study. Fourth, our study only recruited male patients with SCZ, and further investigation should also be performed to examine whether there was an interrelationship between glucose metabolism and gonadal hormones among female patients.

## CONCLUSION

This study demonstrates that male DNFES patients had abnormal glucose metabolism parameters and gonadal hormone levels. Fasting insulin was negatively associated with levels of TESTO in men, suggesting a close relationship between glucose metabolism and hormones in male patients at the onset of SCZ. Our study focused on the interrelationship between metabolic and endocrine signaling pathways related to SCZ, providing important translational insights into the complex and multifaceted pathophysiological mechanism of SCZ. However, considering that these biomarkers are easily influenced by other demographic and clinical confounders, as well as our cross-sectional design, we did not draw a causal conclusion about the relationship between glucose metabolism and sex hormone levels and further studies with a longitudinal design are warranted to better understand the impact of sex hormones on metabolic markers in the early stage of SCZ.

## Figures and Tables

**Table 1:** The demographic, glucose metabolism parameters, and sex hormone levels in patients with schizophrenia.

**-**	**Male Patients** **n = 99**	**Normal Reference Range**
Age(ys), median (IQR)	23.00(19.00,30.00)	-
Course(ms), median (IQR)	24.00(6.00,51.00)	-
Onset age(ys), median (IQR)	20.92(17.33,28.00)	-
Waist(cm), median (IQR)	79.9(76.6,86.6)	< 85
BMI(kg/m^2^), mean ± SD	23.14 ± 3.34	18.5-23.9
Education(ys), median (IQR)	12.00(10.00,15.00)	-
FBG, mean ± SD	5.23 ± 0.36	3.9-6.1
FINS, median (IQR)	9.71(5.80,13.97)	2.6-24.9
HOMA-IR, median (IQR)	2.36(1.37,3.18)	< 2.69
HbA1c(%), mean ± SD	5.27 ± 0.31	4.8-5.9
E2, median (IQR)	103.00(78.00,124.00)	73-173
FSH, median (IQR)	3.60(2.60,5.30)	1.27-19.26
PROG, median (IQR)	1.81(1.14,2.40)	0.45-6.55
LH, median (IQR)	3.60(2.55,4.82)	1.24-8.62
TESTO, mean ± SD	13.79 ± 4.62	6.07-27.1

**Abbreviations:** ys years, ms months, BMI body mass index, FBG fasting blood glucose, FINS fasting insulin, HOMA-IR insulin resistance index, E2 estradiol, FSH follicular-stimulating hormone, PROG progesterone, LH luteinizing hormone, and TESTO testosterone, PRL prolactin.

**Table 2:** Associations between glucose metabolism parameters and sex hormones in male patients.

-	-	E2	FSH	PROG	LH	TESTO
FBG	r	-0.067	-0.020	0.002	-0.063	-0.225
	p	0.513	0.846	0.981	0.533	0.025
HbA1c	r	-0.118	-0.104	-0.081	-0.010	0.024
	p	0.245	0.308	0.423	0.923	0.812
FINS	r	0.000	0.062	-0.288	0.107	-0.340
	p	0.997	0.541	0.004	0.290	<0.001
HOMA-IR	r	-0.012	0.062	-0.280	0.090	-0.351
	p	0.910	0.543	0.005	0.374	<0.001

**Abbreviations:** FBG fasting blood glucose, HbA1c glycated hemoglobin, FINS Fasting serum insulin, HOMA-IR insulin resistance index, E2 estradiol, FSH follicular-stimulating hormone, PROG progesterone, LH luteinizing hormone, and TESTO testosterone.

**Table 3:** Linear regression of the factors related to fasting insulin in male patients with schizophrenia.

**Sex**	**-**	**Unstandardized Coefficients**		**Standardized Coefficients**	***t***	***p***
		**B**	**Sth. Error**	**Beta**		
Male	(Constant)	-5.476	6.022	-	-0.909	0.366
	PROG	-0.518	0.614	-0.081	-0.844	0.401
	TESTO	-0.283	0.129	-0.208	-2.192	0.031
	Waist	4.995	3.151	0.216	1.585	0.116
	BMI	0.389	0.258	0.207	1.503	0.136

**Abbreviations:** E2 estradiol, PROG progesterone, BMI body mass index, and TESTO testosterone.

## Data Availability

The data that support the findings of this study are available from the corresponding author [XL], upon reasonable request.

## LIST OF ABBREVIATIONS

DNFES: Drug-naïve First-episode Schizophrenia Patients
FSH: Follicular-stimulating Hormone
IQRs: Interquartile Ranges
LH: Luteinizing Hormone
PANSS: Positive and Negative Syndrome Scales
SCZ: Schizophrenia
