# Supplementary material for: Glucose Metabolism and Sex Hormones in Male Patients with Medication-naïve First-episode Schizophrenia: A Large-scale Cross-sectional Study
Source: Curr Neuropharmacol. 2024 Feb 16;22(13):2263–70. doi: 10.2174/1570159X22666240212141602 (PMC11337684; doi:10.2174/1570159X22666240212141602)
Supplement: Supplementary file 1 — Supplementary material is available on the publisher’s website along with the published article. [file CN-22-2263_SD1.pdf]

Supplementary Material

Glucose Metabolism and Sex Hormones in Male Patients with Medication-naïve First-episode Schizophrenia: A Large-scale Cross-sectional Study

Meihong Xiu[1], Meng Hao[1], Cai Liu[1], Maodi Sun[2] and Xiaoe Lang[3,*]

[1]Peking University HuiLongGuan Clinical Medical School, Beijing Huilongguan Hospital, Beijing, China; [2]North University of China, Taiyuan, China; [3]Department of Psychiatry, First Hospital of Shanxi Medical University, Taiyuan, China

Table S1. Kolmogorov-Smirnov One-sample test for the normality of demographic and glucose metabolism data.

		FBG	HbA1c	FINS	HOMA-IR	Age	Onset Age	Course	Waist	BMI	Education Years
N		223	223	223	223	223	223	223	223	223	223
Normal parameter[a,b]	Mean	5.1664	5.2763	11.9791	2.7760	25.96	23.81	25.887	2.344	22.62	11.78
	SD	.38977	.33292	6.22563	1.52714	7.772	7.683	20.748	.264	3.448	3.451
Most Extreme Differences	Absolute	.032	.059	.103	.113	.137	.134	.192	.144	.047	.079
	Positive	.032	.024	.103	.113	.137	.134	.192	.144	.047	.075
	Negative	-.026	-.059	-.070	-.082	-.100	-.081	-.148	-.092	-.036	-.079
Kolmogorov-Smirnov Z			.881	1.537	1.682	2.052	1.998	2.872	2.14	.697	1.179
Asymp. Sig. P (2-tailed)			.420	.018	.007	.000	.001	.000	.000	.717	.124

Abbreviations: BMI body mass index, FBG fasting blood glucose, FINS fasting insulin, HOMA-IR insulin resistance index.

1875-6190/24 $65.00+.00 © 2024 Bentham Science Publishers

Table S2. Kolmogorov-Smirnov One-sample test for the normality of sex hormones in male patients.

		FSH	PROG	LH	TESTO	E2	PRL
N		99	99	99	99	99	99
Normal parameter[a,b]	Mean	4.1525	1.9126	3.9290	13.7889	105.8081	237.0808
	SD	2.06661	.97581	1.95236	4.61625	37.37228	165.58700
Most Extreme Differences	Absolute	.119	.117	.126	.059	.097	.169
	Positive	.119	.117	.126	.059	.095	.169
	Negative	-.084	-.077	-.099	-.030	-.097	-.108
Kolmogorov-Smirnov Z			1.165	1.250	.584	.965	1.684
Asymp. Sig. P (2-tailed)			.132	.088	.885	.309	.007

Abbreviations: E2 estradiol, FSH follicular-stimulating hormone, PROG progesterone, LH luteinizing hormone, and TESTO testosterone.

Table S3. Kolmogorov-Smirnov One-sample test for the normality of sex hormones in female patients.

		FSH	PROG	LH	TESTO	E2	PRL
N		124	124	124	124	124	124
Normal parameter[a,b]	Mean	5.8895	10.4603	8.1656	1.8782	337.1210	294.5484
	SD	2.57015	13.58997	8.82126	.77957	254.80299	203.43643
Most Extreme Differences	Absolute	.056	.301	.205	.069	.182	.144
	Positive	.056	.301	.205	.059	.182	.144
	Negative	-.043	-.221	-.189	-.069	-.117	-.113
Kolmogorov-Smirnov Z		.621	3.349	2.286	.765	2.021	1.602
Asymp. Sig. P (2-tailed)		.835	.000	.000	.603	.001	.012

Abbreviations: E2 estradiol, FSH follicular-stimulating hormone, PROG progesterone, LH luteinizing hormone, and TESTO testosterone.
